# Formulation and Evaluation of Nano Lipid Carrier-Based Ocular Gel System: Optimization to Antibacterial Activity

**DOI:** 10.3390/gels8050255

**Published:** 2022-04-21

**Authors:** Sadaf Jamal Gilani, May Nasser bin Jumah, Ameeduzzafar Zafar, Syed Sarim Imam, Mohd Yasir, Mohammad Khalid, Sultan Alshehri, Mohammed M. Ghuneim, Fatima M. Albohairy

**Affiliations:** 1 Department of Basic Health Sciences, Preparatory Year, Princess Nourah bint Abdulrahman University, Riyadh 11671, Saudi Arabia; sjglani@pnu.edu.sa; 2 Biology Department, College of Science, Princess Nourah bint Abdulrahman University, Riyadh 11671, Saudi Arabia; mnbinjumah@pnu.edu.sa; 3 Environment and Biomaterial Unit, Health Sciences Research Center, Princess Nourah bint Abdulrahman University, Riyadh 11671, Saudi Arabia; 4 Saudi Society for Applied Science, Princess Nourah bint Abdulrahman University, Riyadh 11671, Saudi Arabia; 5 Department of Pharmaceutics, College of Pharmacy, Jouf University, Sakaka 72341, Saudi Arabia; 6 Department of Pharmaceutics, College of Pharmacy, King Saud University, Riyadh 11451, Saudi Arabia; salshehri1@ksu.edu.sa; 7 Department of Pharmacy, College of Health Science, Arsi University, Asella 396, Ethiopia; mohdyasir31@gmail.com; 8 Department of Pharmacognosy, College of Pharmacy, Prince Sattam Bin Abdulaziz University, Al-Kharj 11942, Saudi Arabia; drkhalid8811@gmail.com; 9 Department of Pharmacy Practice, College of Pharmacy, AlMaarefa University, Ad Diriyah 13713, Saudi Arabia; mghoneim@mcst.edu.sa; 10 Electron Microscope Research Unit, Health Sciences Research Center, Princes Nourah bint Abdulrahman University, Riyadh 11671, Saudi Arabia; fmalbohairy@pnu.edu.sa

**Keywords:** NLs, in situ gel, ocular delivery, azithromycin, antibacterial, HET CAM

## Abstract

The present research work was designed to prepare Azithromycin (AM)-loaded nano lipid carriers (NLs) for ocular delivery. NLs were prepared by the emulsification–homogenization method and further optimized by the Box Behnken design. AM-NLs were optimized using the independent constraints of homogenization speed (A), surfactant concentration (B), and lipid concentration (C) to obtain optimal NLs (AM-NLop). The selected AM-NLop was further converted into a sol-gel system using a mucoadhesive polymer blend of sodium alginate and hydroxyl propyl methyl cellulose (AM-NLopIG). The sol-gel system was further characterized for drug release, permeation, hydration, irritation, histopathology, and antibacterial activity. The prepared NLs showed nano-metric size particles (154.7 ± 7.3 to 352.2 ± 15.8 nm) with high encapsulation efficiency (48.8 ± 1.1 to 80.9 ± 2.9%). AM-NLopIG showed a more prolonged drug release (98.6 ± 4.6% in 24 h) than the eye drop (99.4 ± 5.3% in 3 h). The ex vivo permeation result depicted AM-NLopIG, AM-IG, and eye drop. AM-NLopIG exhibited significant higher AM permeation (60.7 ± 4.1%) than AM-IG (33.46 ± 3.04%) and eye drop (23.3 ± 3.7%). The corneal hydration was found to be 76.45%, which is within the standard limit. The histopathology and HET-CAM results revealed that the prepared formulation is safe for ocular use. The antibacterial study revealed enhanced activity from the AM-NLopIG.

## 1. Introduction

The treatment of ocular diseases is challenging due to the typical anatomy and physiology of the eye [1]. To attain the required amount of drug at the site of action at an appropriate time is difficult due to the dilution of formulations by tear fluid, lachrymal fluid, eyelid blinking, tear fluid turnover, and nasolacrimal drainage. Most ocular diseases are treated by eye drops, but only 5% of an administered dose is available for therapeutic action. The remaining dose was excreted out by various protection mechanisms. Therefore, the bioavailability of eye drops is low and they require multiple and frequent dosing, which may produce side effects [2,3].

The administration of topical non-invasive ocular formulations is the more preferable way to treat the anterior segment of the eye. To enhance the ocular bioavailability of the drugs, various novel drug carriers were investigated by the researchers. The delivery systems include thymoquinone liposomes [4], pilocarpine niosomes [5], acetonide polymeric NPs [6], clarithromycin SLNs [7], methazolamide NLCs [8], besifloxacin nanoemulsion [9], and tacrolimus in situ gel system [10].

Among these nano-delivery systems, NLCs have been widely used as potential carriers belonging to lipid base nano-system [11]. NLCs are nano-metric size particles and give high entrapment efficiency due to lesser crystallinity of the lipid at low surfactant concentrations [12]. It also increases corneal permeability and bioavailability as well as reduces the local and systemic side effects. Various research studies have investigated NLCs for the treatment of ocular diseases [13,14]. Lakhani et al. formulated amphotericin B-loaded NLCs for ocular delivery and exhibited higher entrapment efficiency and higher antifungal activity than the marketed formulation [15]. In another study, Kiss et al. prepared dexamethasone NLCs for ocular inflammation. The prepared formulations exhibited a significant drug concentration in the stroma layer, confirmed by the porcine cornea study [16]. The application of NLCs was further explored by Seyfoddin and his research team and they developed acyclovir NLCs that exhibited higher entrapment efficiency than solid lipid nanoparticles as well as depicted high and faster permeation across the corneal membrane [17]. Besifloxacin-loaded NLC formulations were evaluated for different parameters [18]. The prepared formulation showed nano-metric size, high entrapment efficiency, and significant enhancement in corneal permeation.

Due to the low viscosity of the NLC formulations, their efficacy can be enhanced by transforming them into a sol-gel system. It is prepared in solution form and changes to gel phase in a cul-de-sac by temperature, ion, and pH [19]. Due to conversion into the gel phase, the increase in corneal residence time takes place. It leads to decreased dosing frequency and increases therapeutic efficacy and may reduce the systemic side effects by minimizing the nasolacrimal outflow. Different research designs were reported for the NLC-based sol-gel system by the different administration routes. Ciprofloxacin-loaded NLC in situ gel was prepared and evaluated for the different parameters. The prepared formulations depicted 3.5-fold and 1.9-fold enhancements in the flux and permeation compared to the marketed ciprofloxacin formulation [20]. In another study, moxifloxacin NLC-laden in situ gel was developed and evaluated for endophthalmitis infection. The formulation exhibited a twofold higher permeation than pure drug solution [21]. Natamycin-loaded NLC in situ gel was prepared and optimized by the factorial design method. The formulations were prepared using guar gum, boric acid, and Carbopol^®^ 940 as a gelling agent. The prepared formulations depicted lower flux value in comparison to Natacyn^®^ suspension [22].

Alginates are natural, nontoxic, mucoadhesive, viscosity enhancing polymers derived from different brown seaweeds [23]. They are used for ocular in situ gel systems due to their mucoadhesive, biocompatible, and non-toxic nature [24,25]. They have the properties of effective gelling capacity and viscosity and are commonly used in in situ gelling systems [26]. The prepared ocular solution has a low viscosity at room temperature for easy administration and interacts with the calcium ions in the tear fluid to form a gel matrix and lead to controlled drug release [27]. The alginates interact with divalent cations and bind to the guluronate blocks of the sodium alginate to facilitate gelation. The cross-linking of the cation–guluronate blocks then yields a gel-like structure, with a slow and steady drug release at the application site [28]. Draize ocular irritation studies on rabbits also reported sodium alginates to be nonirritating to the eye [23,29]. Hydroxypropyl methylcellulose (HPMC) is added into the formulation as a viscosity enhancer. It is a non-ionic, nontoxic, viscoelastic polymer with good swelling capacity [27,30].

Azithromycin (AM) is a semisynthetic macrolide antibiotic. It is a broad-spectrum antibiotic and is highly stable in an acidic environment compared to other macrolides. It acts by inhibiting the 50s ribosomal subunit of bacteria and inhibits protein synthesis. It is commercially available on the market as eye drops.

The present research work was designed with two steps. In the first step, AM-NLs were prepared by the emulsification homogenization method and further optimized by the Box Behnken design (BBD) using different independent variables. BBD-based optimized AM-NLop was further converted into an in situ gel (sol-gel) system by using a thermosensitive gelling agent. The in situ gel was further characterized for viscosity, gelling capacity, in vitro release, ex vivo corneal permeation, ocular tolerance, and antimicrobial evaluation.

## 2. Result and Discussion

### 2.1. Screening of Solid and Liquid Lipids

**Figure 1:**
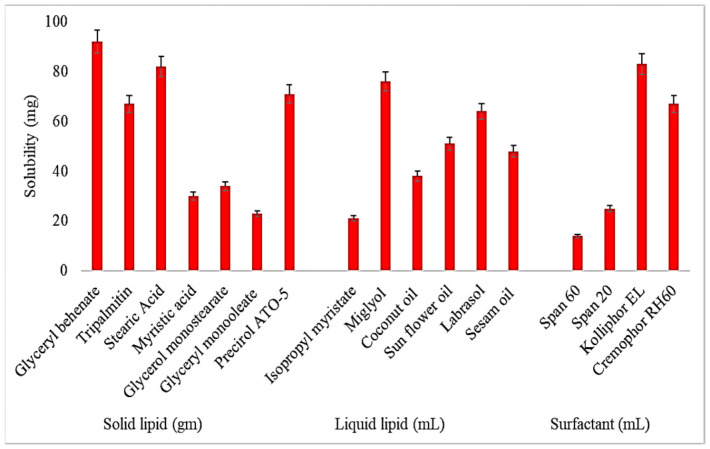
Solubility profile of Azithromycin in different lipids (solid and liquid) and surfactants. Study performed in triplicate and results shown as mean ± SD.

Screening of the solid lipids, liquid lipids, and surfactants used for the development of NLs was performed based on the maximum solubility of AM, and the results are expressed in Figure 1. The order of solubility of AM in various solid lipids is Glyceryl behenate > Stearic acid > Precirol ATO-5 > Tripalmitin > Glycerol monostearate > Myristic acid > Glyceryl monooleate. The highest solubility of AM was found in GB (92.8 ± 6.2 mg/g), and this was selected for further use as a solid lipid. In the case of liquid lipids, the order of solubility of AM was found as follows: Miglyol > Labrasol > Sunflower oil > Sesame oil > Coconut oil > Isopropyl myristate. The maximum solubility of AM was found to be in Miglyol (76.3 ± 4.8 mg/mL), and this was selected as the optimal liquid lipid. The order of solubility of AM in various surfactants is Kolliphor EL > Cremophor RH60 > Span 20 > Span 60. The highest solubility of AM was found to be in Kolliphor EL (83.1 ± 4.5 mg/mL). Based on the maximum solubility of AM, Glyceryl behenate, Miglyol, and Kolliphor EL were used for the formulation of NLs.

### 2.2. Screening of Miscibility Ratio of Solid and Liquid Lipid

The miscibility ratio (solid and liquid lipid) was determined by mixing the melted solid and liquid lipid in different ratios, i.e., 9:1, 8:2, 7:3, 6:4, 5:5, and 2:8. Among the tested ratios, the combination 6:4 ratio was found to be the best blend because it did not show any phase separation, oil droplets, or crystallization. So, this 6:4 ratio of solid and liquid lipids was selected for the development of NLs.

### 2.3. Optimization

The Box Behnken design (BBD) depicted a total of seventeen compositions (five common) using the independent variables of homogenization speed (A), surfactant concentration (B), and lipid concentration (C) at three different levels (low, medium, high), as shown in Table 1. The findings of the prepared AM-NLs were added to the software to obtain the optimized composition of independent constraints. Their effects were observed on the different models to obtain an optimized combination with desirable particle size and encapsulation efficiency. The impact of homogenization speed (A), surfactant concentration (B), and lipid concentration (C) was chosen based on a non-significant lack of fit and low predicted residual error sum of squares value (PRESS value) (Table 2). The polynomial equations were generated by ANOVA to explore the effect of homogenization speed (A), surfactant concentration (B), and lipid concentration (C) on responses (particle size and encapsulation efficiency). The positive and negative value of the polynomial equation indicates the synergistic and antagonistic effect on the dependent factors (responses). Among the different models of the responses, namely particle size (Y_1_) and entrapment efficiency (Y_2_), the quadratic model was found to be the best fitting model [16].

### 2.4. Influence of Independent Variables on Particle Size (Y_1_)

**Figure 2:**
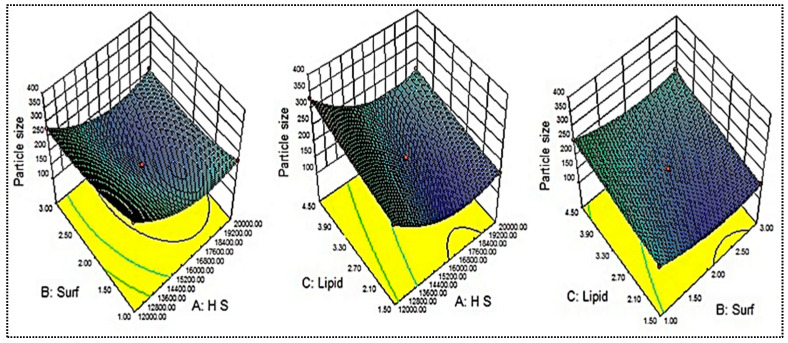
Effect of independent variables (Homogenization speed—A, Surfactant—B, Lipid—C) on the particle size (Y_1_).

The mean particle size of the prepared AM-NLs was between 154.7 ± 7.3 (AM-NL10) and 352.2 ± 15.8 nm (AM-NL1), as shown in Table 1. The minimum particle size (Y_1_) was found from the formulation (AM-NL10) prepared with homogenization speed of 16,000 rpm, surfactant concentration of 3% *w/v*, and lipid concentration of 1.5% *w/v*. The maximum particle size (Y_1_) was found from the formulation (AM-NL1) prepared with composition homogenization speed of 12,000 rpm, surfactant concentration of 1% *w/v*, and lipid concentration of 3% *w/v*. From the results, there is a significant difference in the particle size was observed by changing the composition of independent variables. The effects of independent variables (homogenization speed, surfactant concentration, and lipid concentration) were evaluated on the 3D response surface plot (Figure 2). As shown in Figure 2, homogenization speed (A) showed a dual effect on particle size. The increase in the homogenization speed from a low level to a high level caused the particle size to decrease. After reaching an intermediate level, the further increase in the homogenization speeds results in increased particle size. The increase in size may take place due to the aggregation of particles at a higher homogenization speed. The small globules of emulsion aggregate in the inter-particular space of big globules, leading to the re-coalescence or Ostwald ripening. Besides this, surfactant (B) was responsible for the decrease in interfacial tension, and as per the Laplaces pressure theory, as the interfacial tension decrease, more droplets are disrupted into fine particles. Hence, the surfactant concentration exhibited a negative effect on particle size. Similar results are reported in the literature [21]. At a fixed homogenization speed (A) and surfactant concentration (B), the particle size (Y_1_) increases with increased lipid concentration. This might be due to an enhancement in the viscosity of lipid dispersion [31]. Moreover, there was a high chance of incomplete emulsification at a high lipid concentration and low surfactant concentration (due to lack of surfactant), which was responsible for the aggregation of lipid nanoparticles. The controlled optimal particle size is good for the stability of nano-dispersion as it promotes the Brownian motion and prevents sedimentation [18].

The effect of independent variables was further statistically analyzed by the polynomial Equation (1).

Particle size (Y_1_) = +178.2 − 50.27A − 21.8B + 30.48C + 12.75AB − 3.90AC + 3.80BC + 62.24A^2^ + 27.99B^2^ + 3.94C^2^ (1)

The above equation states that homogenization speed (A, coefficient −50.27) and surfactant (B, coefficient −21.80) showed a negative effect. On the other hand, the third variable, lipid concentration (C, coefficient +30.48), depicted a positive effect. The quadratic coefficient of A^2^, B^2^, and C^2^ showed the positive values of +62.24, 27.99, and +3.94, respectively. The interpretation explains that the variables alone and in combination had a positive effect on the particle size. Based on insignificant lack of fit (F = 0.61, P = 0.6419) and the lowest precision (313.33) value, the quadratic model was chosen as the best fitting model to describe the influence of independent variables on particle size.

### 2.5. Influence of Independent Variables on Entrapment Efficiency (Y_2_)

**Figure 3:**
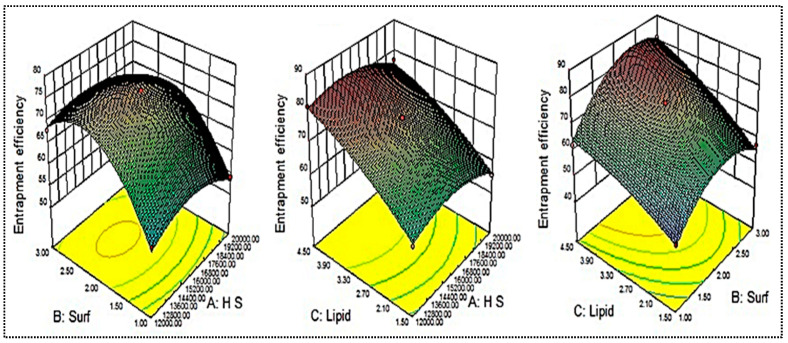
Effects of independent variables (Homogenization speed—A, Surfactant—B, Lipid—C) on the encapsulation efficiency (Y_2_).

The mean entrapment efficiency of the prepared AM-NLs was found between 48.8 ± 1.1 (AM-NL9) and 80.9 ± 2.9 (AM-NL12), as shown in Table 1. The minimum entrapment efficiency (Y_2_) was found from the formulation (AM-NL9) prepared with homogenization speed of 16,000 rpm, surfactant concentration of 1% *w/v*, and lipid concentration of 1.5% *w/v*. The maximum particle size (Y_1_) was found from the formulation (AM-NL12) prepared with composition homogenization speed of 16,000 rpm, surfactant concentration of 3% *w/v*, and lipid concentration of 4.5% *w/v*. From the results, a significant difference in the entrapment efficiency was observed by changing the composition of independent variables. The effects of independent variables (homogenization speed, surfactant concentration, and lipid concentration) were evaluated on the 3D response surface plot (Figure 3). As shown in Figure 3, homogenization speed (A) showed a synergistic effect on entrapment efficiency. With the increase in the homogenization speed from a low level (12,000 rpm) to an intermediate level (16,000 rpm), AM entrapment increased. After reaching the intermediate level, the further increase in the homogenization speed decreased the entrapment efficiency. The decrease in entrapment efficiency may be due to the leaching of drugs at a high homogenization speed. The surfactant (B) also showed a dual effect on the entrapment efficiency. The increase in surfactant concentration led to a decrease in interfacial tension and a greater amount of solubilized and entrapped AM. The presence of a sufficient surfactant concentration prevents the drug expulsion and ultimately forms stable nanoparticles. Contrarily, if sufficient lipids are not available for drug entrapment, the reverse results will occur [32]. The third variable, lipid concentration (C), depicted a positive effect on entrapment efficiency. The increase in lipid content resulted in a greater amount of AM entrapped inside the NLs. The possible reason for this is the availability of more space for the accommodation of drugs within the NL vesicles [33]. A low concentration of surfactants and high concentration of lipids gives lesser entrapment due to incomplete emulsification.

The effect of independent variables was further evaluated by the polynomial equation given below:

Entrapment efficiency = +74.71 − 1.62A + 5.98B + 10.13C + 0.70AB − 0.40AC + 2.70BC − 4.42A^2^ − 10.02B^2^ − 2.51C^2^ (2)

As depicted in Equation (2), homogenization speed with the magnitudes –1.62 (A) and −4.42 (A^2^) exhibited a negative effect on entrapment efficiency. On the other hand, upon increasing the surfactant concentration (magnitude +5.98), the entrapment efficiency was increased if sufficient lipids were present in the dispersion. This might be due to the solubility of the drug in the presence of sufficient surfactants. The quadratic model was considered as the best fitting model based on non-significant lack of fit (F = 3.49, *p*-value 0.1295) and lowest precision (172.2) value (Table 2). It described the influence of independent variables on entrapment efficiency.

### 2.6. Optimized Composition

Table 2 and Figure 2 and Figure 3 indicate that all the process and formulation variables were well controlled, and the obtained values were in an acceptable range. Based on the above results, it was concluded that all three variables had a significant effect on the responses. Among the 17 prepared formulations, AM-NL13 was selected for further point prediction optimization by slightly changing the composition and further evaluated for particle size and entrapment efficiency (Table 3). The practical results of particle size and entrapment efficiency were found to be closer to each other, and the overall desirability was found to be 0.992, which confirms the validity of the model.

**Figure 4:**
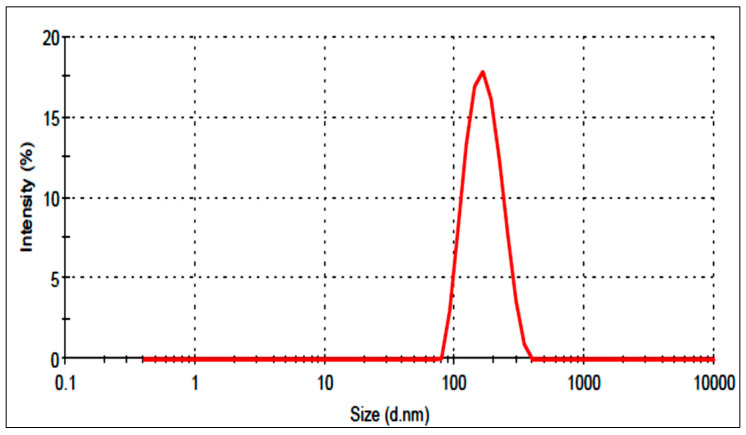
Particle size distribution image of Azithromycin-loaded nano lipid carrier (AM-NLop).

The selected optimized formulation (AM-NLop) was the composition prepared with homogenization speed of 16,500 rpm, surfactant concentration of 2.25% *w/v*, and lipid concentration of 3% *w/v*. It showed particle size and entrapment efficiency of 154.1 ± 6.35 nm (Figure 4) and 78.15 ± 1.9%. It depicted a PDI value of 0.18 and negative zeta potential (−34.24 mV), indicating higher stability. The low PDI (less than 0.5) and high negative zeta potential (±30 mV) revealed the greater homogeneity of particles. The predicted value of particle size and entrapment efficiency was found to be 161.3 nm and 79.8%, and the value was found to be closer to the predicted values.

### 2.7. X-ray Diffraction (XRD) Analysis

**Figure 5:**
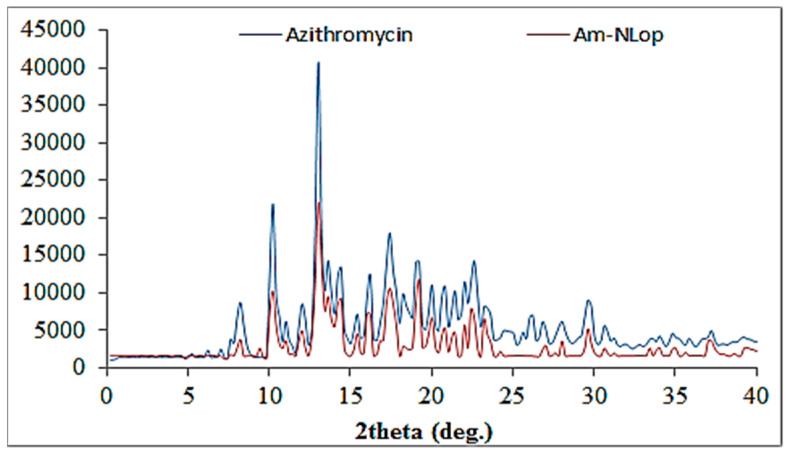
XRD image of Azithromycin and Azithromycin-loaded nano lipid carrier (AM-NLop).

Figure 5 shows the diffractogram of AM and AM-NLop. AM exhibited a characteristic highly intense peak at 2-theta levels of 8.2°, 10.2°, 13°, 16.2°, 17.2°, 19.1°, and 29.6°, assuring its crystallinity. The diffractogram of AM-NLop did not exhibit any characteristic sharp peak of AM after encapsulating into NLs. The low intensity and broad AM peaks indicate that AM was encapsulated or solubilized in a lipid matrix. The reduction in the intensities takes place due to some modification in the crystallinity in the NLs, attributed to the disordering of the solid lipid crystalline structure due to the presence of liquid lipids [34].

### 2.8. Gel Evaluation

#### 2.8.1. Clarity and Gelling Ability

The formulation was found to be clear upon observation with a black and white background. The presence of foreign particles may produce irritation to the soft tissue of the ocular region. It was also evaluated for sol to gel properties after gradually converting into a gel after the addition of the sol form into STF. This gelling is due to the replacement of sodium from sodium alginate with Ca^+2^ in tear fluid, forming calcium alginate. Table 4 shows the viscosity results of AM-NLopG and AM-G formulations. The viscosity of AM-NLopG in the solution state was found to be very low, whereas after conversion into gel form, a significant enhancement in the viscosity was observed. The viscosity was also evaluated for the control formulation AM-IG. In solution form, it shows the viscosity of 235.34 ± 7.21 cps, and after conversion into gel state, it showed 495.18 ± 8.72 cps. AM-NLopG showed higher viscosity in the solution state due to the presence of different ingredients used to prepare NLs, and after converting into gel state, greater viscosity was also observed due to higher gelling capacity.

#### 2.8.2. Texture Analysis

Texture analysis of AM-NLopIG and AM-IG (control) formulation was performed to determine the mechanical properties, and the results are expressed in Table 4. The data show that cohesiveness, adhesiveness, and hardness of AM-NLopIG were found to be significantly changed (*p* < 0.05) in sol and gel states. It showed cohesiveness of 0.78 ± 0.12 (sol) to 0.86 ± 0.09 (gel), adhesiveness of 14.24 ± 1.03 (sol) to 29.43 ± 1.12 (gel), and hardness of 3.21 ± 0.24 (sol) to 8.76 ± 0.51 (gel). The results of the mechanical properties of AM-NLopG and AM-IG did not show significant changes (*p* > 0.05). Interaction of Ca^+2^ with the gelling polymer in situ gel system directly affects mechanical properties. The higher value of adhesiveness indicates more adhesion to the corneal surface and increases the residence time. However, the cohesiveness of the formulation reduces the irritation and makes it easy to spread on the ocular surface [35].

#### 2.8.3. In Vitro Drug Release

**Figure 6:**
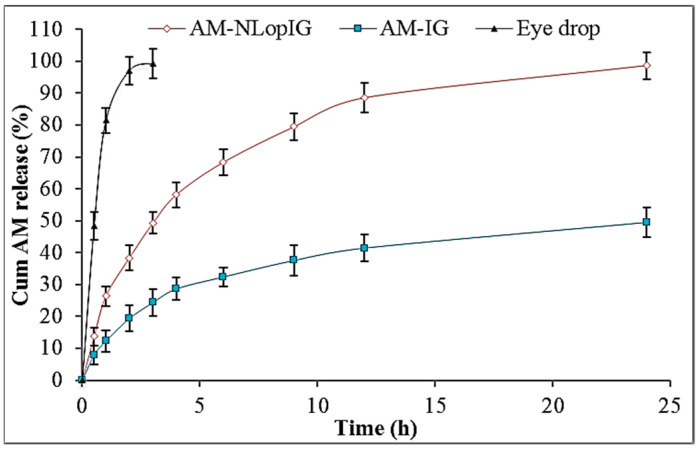
In vitro release data of the Azithromycin nano lipid carrier (AM-NLop), Azithromycin nano lipid carrier-based in situ gel (AM-NLopIG), and AM eye drop. The tests were performed in triplicate and data are shown as mean ± SD.

Figure 6 depicts the comparative release profile of AM-NLopIG, AM-IG, and eye drop using the dialysis bag method. The release of AM from AM-NLopIG and AM-IG was found to be 98.65 ± 4.65% and 49.48 ± 4.23%, respectively, in 24 h. The eye drop depicted 99.45 ± 5.3% release in 3 h. AM-NLopIG exhibited biphasic release behavior, with initial fast release and later slow release. The initial fast release may be due to the nano-sized particles of NLs and a higher effective surface area. The drug particles adsorbed on the surface of NLs enter the dissolution media. Later, the slower drug release was found to be due to the formation of gel matrix, and the drug needs to cross it as well as the dialysis membrane. The eye drop showed quicker release in 3 h due to the lesser viscosity, and the drug is available for release. AM-IG showed significantly less release than AM-NLopIG due to the poor solubility and non-availability of surfactants. The surfactant helps to increase the solubility and lowers the interfacial tension between the two phases.

#### 2.8.4. Ex Vivo Permeation Study

The comparative ex vivo permeation results of AM-NLopIG, AM-IG, and eye drop showed significant differences in permeation (%) and flux. AM-NLopIG exhibited significantly (*p* < 0.05) higher permeation (60.76 ± 4.12%) than AM-IG (33.46 ± 3.04%) and eye drop (23.31 ± 3.76%). The flux of AM-NLopIG was found to be 153.43 µg/h.cm^2^, which is 1.82-fold higher than AM-IG and 2.6-fold higher than the eye drop. The higher permeation of AM from AM-NLopIG was found due to the increase in AM solubility after nano-sizing. The presence of lipids and surfactants of NLs increases the endocytosis through the corneal epithelium and leads to greater permeation across the membrane [21,36]. The sol form of a prepared formulation is converted to gel form and the contact time increases. The increase in contact time and the used polymer helps to open the tight junction of the membrane and gives greater permeation. The apparent permeability coefficients of AM-NLopIG, AM-IG, and eye drop were calculated as 2.4 × 10^−1^ cm/s, 1.3 × 10^−1^ cm/s, and 9.3 × 10^−2^ cm/s, respectively. AM-NLopIG showed an enhancement ratio 1.82-fold higher than AM-IG and 2.61-fold higher than the eye drop.

#### 2.8.5. Corneal Hydration

The ocular hydration study was performed to evaluate the ocular toxicity after keeping the formulation with the cornea. After treatment, the corneal hydration was found to be 76.45%, which is within the standard limit of 76–80% [37]. The results of the study revealed that AM-NLopIG did not produce any toxicity and did not alter the structure of the cornea. There was no damage to the cornea observed externally and internally, which was further confirmed by histopathology examination.

#### 2.8.6. Histopathology

**Figure 7:**
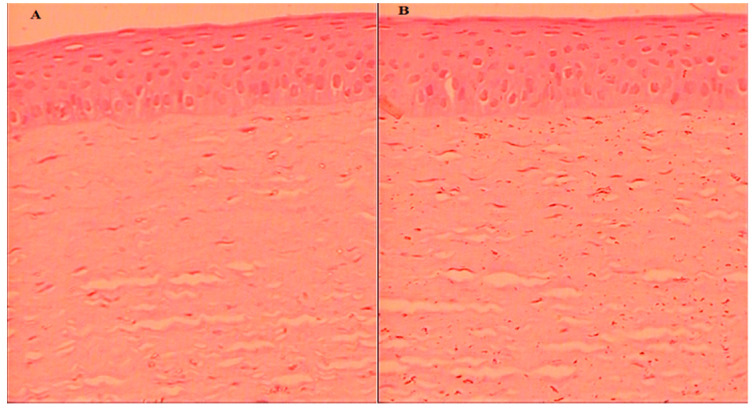
Histopathology image of AM-NLopIG-treated cornea (**A**). NaCl-treated cornea (**B**) taken by light microscope at image scale 40×.

The cornea was collected after the permeation study and assessed for internal damage in the structure. The histopathology of the treated cornea with AM-NLopIG and control (NaCl, 0.9%) was compared to changes in the structure, as depicted in Figure 7A,B. AM-NLopIG-treated cornea revealed no marked change in the internal structure. No damage in cellular structure or histological alterations were observed in the treated cornea, matching with the normal saline-treated cornea. The epithelium, Bowman’s membrane, and stroma of the cornea and the cells were found intact, similar to the control sample. The morphology of the cornea was also well maintained [38]. From the results, we can presume that there is no alteration in the corneal structure, and that AM-NLopIG is nontoxic and compatible with the ocular structure.

#### 2.8.7. HET-CAM Irritation Study

**Figure 8:**
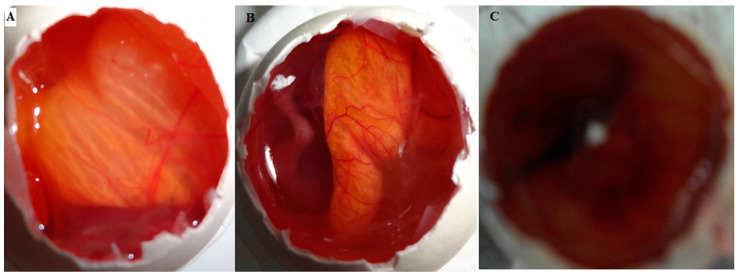
HET-CAM irritation test image treated with AM-NLopIG (**A**), 0.9% NaCl (**B**), and 1% SLS (**C**).

HET-CAM (hen’s egg chorioallantoic membrane) is an in vitro evaluation method. It is helps to determine the irritation potential of formulations and also used as an alternative to the Draize test in rabbits. The results of CAM treated with AM-NLopIG, a negative control (0.9% NaCl), and a positive control (1% SLS) are expressed in Figure 8A–C. AM-NLopIG and the negative control did not exhibit any damage to CAM (blood capillaries). The score was found to be closer to zero and considered as non-irritant. It revealed no damage to the vein or artery, no bleeding and hemorrhage observed. The positive control depicted a high irritant cumulative score of 22.33 (severe irritant) with bleeding and excessive hemorrhage. From the results, it can be concluded that the prepared formulation AM-NLopIG is safe and non-irritant for ocular administration.

#### 2.8.8. Isotonicity Study

**Figure 9:**
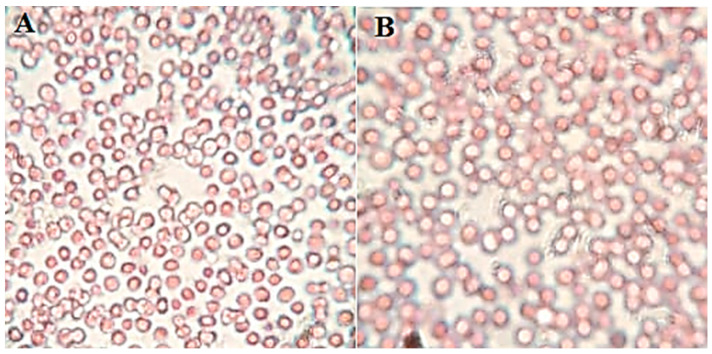
Isotonicity evaluation image of (**A**) AM-NLopIG and 0.9% (**B**) NaCl.

The study was performed to evaluate the damage to the blood cells after treatment with AM-NLopIG. It was evaluated by treating with goat blood and observed under a microscope for any damage to RBCs. AM-NLopIG- and control (0.9% NaCl)-treated blood samples were evaluated for shrinkage and swelling to the blood cells and did not show any damage in RBC, revealing that the formulation is isotonic (Figure 9A,B). The results of the study are supported by the findings of the HET-CAM results. It also revealed no damage to the CAM.

#### 2.8.9. Antimicrobial Study

**Figure 10:**
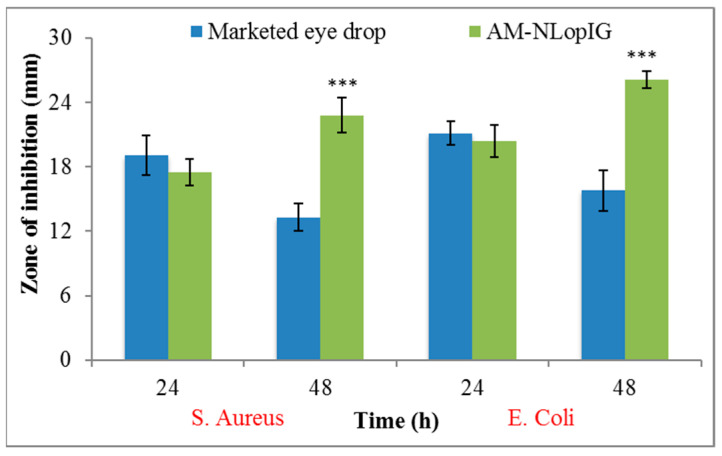
Antimicrobial graph of AM-NLopIG and eye drop evaluated against *S. aureus* and *E. coli*. Results shown as mean ± SD. *** indicates significant difference to the marketed eye drop.

The antimicrobial activity of AM-NLopIG and eye drop was determined by the cup plate method against *S. aureus* and *E. coli* (Figure 10). AM-NLopIG depicted ZOI of 17.5 ± 1.7 mm and 20.4 ± 2.1 mm against *S. aureus* and *E. coli* at 24 h. The same formulation was further evaluated at 48 h and there was a significant (*p* < 0.05) enhancement in ZOI. It showed a ZOI of 22.8 ± 2.2 mm and 26.1 ± 1.9 mm against *S. aureus* and *E. coli* at 48 h. The eye drop showed less antibacterial activity against *S. aureus* and *E. coli* as 19.1 ± 1.4 mm and 21.1 ± 2.2 mm, respectively, at 24 h. Significantly less activity was observed at 48 h. It showed ZOI of 13.3 ± 1.2 mm and 15.8 ± 1.7 mm. The prepared formulation AM-NLopIG showed significantly (*p* < 0.05) higher susceptibility (1.4-fold and 1.5-fold) than the eye drop. The enhanced antibacterial activity is due to the nano-sized particles and high surface energy. It will have a large surface area to act on the cell wall of microorganisms and increase the membrane permeability of the bacteria due to more enhanced mucoadhesive properties than the eye drop.

## 3. Conclusions

An Azithromycin-loaded nano lipid carrier was prepared by the emulsification–homogenization method. The formulations were optimized by BBD design using homogenization speed (A), surfactant concentration (B), and lipid concentration (C) as independent variables. The prepared formulations showed nano-metric size and high encapsulation efficiency. The optimized formulation (AM-NLop) showed a nanoparticle size of 154.1 ± 6.35 nm, entrapment efficiency of 78.15 ± 1.9%, a PDI value of 0.18, and negative zeta potential (−34.24 mV). It was further converted into the sol-gel system to enhance the mucoadhesion. It showed optimal physicochemical properties in the solution as well as in gel form. The release and permeation study results depicted prolonged drug release as well as enhanced permeation (1.82-fold higher than AM-IG and 2.61-fold higher than eye drop). AM-NLopIG was found to be isotonic as well as non-irritant (HET CAM and histopathology). The antibacterial study results revealed greater activity (1.4-fold and 1.5-fold) against both organisms. From the results, it can be concluded that the prepared AM-NLs and AM-NL-based sol-gel system is an ideal delivery system to treat ocular diseases.

## 4. Materials

Azithromycin (AM) was procured from Alembic Pharmaceutical Ltd. (Ahmedabad, India). Glyceryl behenate (GB, melting point ~70 °C), Labrasol, and Precirol ATO-5 were procured from Gattifosse (Mumbai, India). Tripalmitins, Stearic acid, Myristic acid, Glycerol monostearate, Glyceryl monooleate, methanol, and acetonitrile were procured from Sigma-Aldrich (St. Louis, MO, USA). Isopropyl myristate, Miglyol, Coconut oil, Sunflower oil, Labrasol, and Sesame oil were procured from Loba Chemie (Mumbai, India). The Span 60, Span 20, Kolliphor EL, and Cremophor RH60 were procured from Acros organic (Somerset, NJ, USA). Zaha eye drops (Azithromycin eye solution, 1% *w/v*, Ajanta Pharma, India) were purchased from a local pharmacy.

## 5. Experimental

### 5.1. Screening of Lipids and Surfactant

The screening of lipids was performed by the solubility of AM in the different solid lipids for the formulation of NLs. The excess amount of AM was added to each melted solid lipid (Glyceryl behenate, Tripalmitin (~64 °C), Stearic acid (~69 °C), Myristic acid (~52 °C), Glycerol monostearate (~74 °C), Glyceryl monooleate (~40 °C), Precirol ATO-5 (~55 °C)) in a glass vial. Similarly, the excess amount of AM was added to liquid lipids (Isopropyl myristate, Miglyol, Coconut oil, Sunflower oil, Labrasol, Sesame oil) and surfactants (Span 60, Span 20, Kolliphor EL, Cremophor RH60) in a glass vial. The samples were vortexed and kept in a water bath shaker (JULABO, Izumi Osaka, Japan) for 72 h. The mixture was centrifuged at 5000 rpm for 30 min, and then AM in each solid lipid was determined by a UV spectrophotometer (Genesys 10S UV-Vis, Thermo Scientific, Waltham, MA, USA) after appropriate dilution.

### 5.2. Selection of Solid and Liquid Lipid Ratio

The melted solid and liquid lipids in different ratios (9:1, 8:2, 7:3, 6:4, 5:5, 2:8) were mixed with continuous stirring. The mixture was cooled, and the smear of lipids was prepared over filter paper. The separation of oil droplets over the filter paper was visually observed.

### 5.3. Optimization

Design Expert software (Design Expert, version 8.0.6, State-Ease Inc., Minneapolis, MN, USA) was used for the optimization [39]. The purpose of this design was to examine the level of independent constraints, namely, homogenization speed (A, 12,000–20,000 rpm), surfactant concentration (B, 1.5–4.5%), and lipid concentration (1.5–4.5%), to achieve the desired size and entrapment efficiency. The polynomial Equation (3) was used to evaluate the statistical assessment of different independent variables on dependent constraints and can be given as:

Y = b_0_ + b_1_A + b_2_B + b_3_ C + b_12_AB + b_13_AC + b_23_BC + b_11_A^2^ + b_22_B^2^ + b_33_C^2^ (3)

where Y is the response related to each independent constraint; b_0_ is the intercept; b_1_–b_33_ are the regression coefficients obtained for observed experimental values; and A, B, and C are the coded values of the independent factors. The coefficients b_1_, b_2_, and b_3_ show the individual parameter effect on the responses when two other parameters remain constant. The interaction terms (like b_12_–b_23_) display the response modification when the two factors are simultaneously altered. Based on the preliminary screening, a range of various independent constraints were decided. These values were fitted in the Design Expert software to obtain the composition of 17 formulations including five center points (common composition). The formulations were prepared, and their particle size and encapsulation efficiency data were placed in BBD to derive the predicted values, different polynomial equations, and model graphs. The obtained results were evaluated to examine the effects of independent constraints on dependent factors.

### 5.4. Development of Azithromycin-Loaded Nanostructure Lipid Carrier

AM-NLs were developed by the emulsification–homogenization technique as per a previously reported method with slight modifications [20]. The various batches (AM-NL1–AM-NL17) were prepared as per the given composition of Table 1. The solid lipid was melted at above 5 °C of its melting point and the liquid lipid and was added with continuous stirring to make the homogeneous mixture. The aqueous surfactant solution was prepared in distilled water and heated at the same temperature. The hot aqueous surfactant solution was added to the melted lipid mixture at 15,000 rpm for 15 min to form a coarse primary emulsion. The primary coarse emulsion was further subjected to a homogenizer (T25 digital Ultra-Turrax IKA, Staufen, Germany) for 5 min. The prepared emulsion was cooled to room temperature to form AM-NLs and further stored for evaluation.

## 6. Characterization

### 6.1. Size, PDI, and Zeta Potential

The size, PDI, and zeta potential were analyzed by a zeta sizer (NanoZS90, Malvern Instrument Ltd., Malvern, UK). The diluted NLs (100-fold) were placed in cuvettes, and size and PDI were determined. For zeta potential, the same diluted sample was placed in electrode-containing cuvettes, and then zeta potential was determined.

### 6.2. Entrapment Efficiency (% EE)

The ultracentrifugation method was employed for the determination of EE from AM-NLs. AM-NLs were placed in a centrifugation tube and centrifuged at 15,000 rpm (Remi, cooling centrifuge, Mumbai, India) for 30 min. The supernatant was collected and further diluted to evaluate the AM content by a UV spectrophotometer, and % EE was calculated by the following equation:

(4) $$\%\;\mathrm{EE}=\frac{\mathrm{Total}\;\mathrm{AM}-\mathrm{Free}\;\mathrm{AM}}{\mathrm{Total}\;\mathrm{AM}}\times 100$$

### 6.3. X-ray Diffraction (XRD) Analysis

An XRD instrument (Ultima IV, Rigaku Inc., Tokyo, Japan) was used to determine the crystallinity of AM after encapsulating into AM-NLop. Each sample was filled in an XRD sample holder and scanned between 5° and 60° at the 2-theta level. Diffractograms of both samples were recorded to compare the change in peak height and width.

### 6.4. Formulation of AM-NLop In Situ Gel

The optimized formulation (AM-NL13) based on particle size and encapsulation efficiency was further converted into in situ gel by using mucoadhesive polymers. Weighed quantities of sodium alginate (1.5%, *w/v*) and hydroxyl propyl methyl cellulose (0.5%, *w/v*) were soaked overnight in distilled water. AM-NL13 was dispersed in polymeric solution to form in situ gel dispersion equivalent to 1% AM and pH was adjusted to 6.5.

### 6.5. Clarity and Gelling Ability

The clarity and gelling ability of AM-NLs in situ gel (AM-NLopIG) were checked visually. For gelling strength, AM-NLopIG (50 µL) was added to simulated tear fluid (2 mL) in a vial and gelling time was noted [40].

### 6.6. Viscosity Determination

The viscosity of AM-NLopIG formulation in solution and gel form was measured by a Brookfield viscometer (Fungi Lab, Madrid, Spain) using spindle number LV3 at 50 rpm angular speed. The temperature was fixed at 37 ± 0.5 °C for the study.

### 6.7. Texture Analysis

This study was performed using a texture analyzer (TA.XTplus100C, stable Microsystem, Ltd., Surrey GU7 1YL, Warrington, UK) as per the previous prescribed method [41]. AM-NLopIG was placed into the sample holder and pressed by a 20 mm diameter cylindrical probe (20 s interval, 2 mm depth). The hardness, cohesiveness, and adhesiveness were analyzed, and the results were compared with the eye drop.

### 6.8. In Vitro Drug Release

The comparative release study of AM-NLopIG, AM-IG (control), and eye drop was performed using a dialysis bag (MWCO 12 kDa). The pretreated dialysis bag was filled with each formulation and dipped into STF (250 mL) at a temperature of 37 ± 0.5 ℃. At a fixed time, 2 mL release content was collected and the same volume of fresh STF was added to maintain the uniform release media volume. The released content was filtered and further diluted to measure the absorbance using a UV spectrophotometer.

### 6.9. Trans-Corneal Permeation Study

The study was performed using goat cornea collected from the slaughterhouse. The whole eyeball was collected and stored in NaCl (0.9%) at 4 °C. The cornea was removed from the eyeball with the sclera and washed. Simulated tear fluid (STF) was used as permeation media and filled into the receptor compartment of the diffusion cell (area 0.6 cm^2^, volume 10 mL). The cornea was mounted between the donor and acceptor compartment and the temperature was maintained at 37 ± 0.5 °C. AM-NLopIG, AM-IG, and eye drop (2 mg of the AM, in percentage) were filled into the donor compartment, and at a fixed time point, the released content (1 mL) was removed. The blank fresh STF was replaced into a diffusion cell to keep a uniform volume. The collected release content was filtered and further diluted for evaluation by the previously validated HPLC method [42]. The HPLC method was performed using mobile phase composition ammonium acetate buffer (0.05M and pH 8) and acetonitrile (60:40 *v*/*v*). The study was performed at a flow rate of 1 mL/min with an injection volume of 20 µL. The % permeation and flux were calculated.

(5) $$\mathrm{Flux}=\frac{\mathrm{Concentration}}{\mathrm{Permeation}\;\mathrm{area}\times \mathrm{ime}}$$

(6) $$\mathrm{Drug}\;\mathrm{Permeation}=\frac{\mathrm{Concentration}\;}{\mathrm{applied}\;\mathrm{area}\;}\times \mathrm{dilution}$$

### 6.10. Corneal Hydration

The corneal hydration test was performed to evaluate the hydration of the cornea after the treatment with AM-NLopIG. The cornea was collected after the permeation study and the initial weight was noted (wet weight). The cornea was placed into the oven (Thermo Scientific, Osterode, Germany) at 80 °C for drying. The cornea was removed from the oven and reweighed to note the dry weight. The % corneal hydration (CH) was calculated using the formula as reported by Mudgil et al. [43].

(7) $$\mathrm{CH}\;\left(\%\right)=\frac{\mathrm{Wet}\;\mathrm{weight}\;-\mathrm{Dry}\;\mathrm{weight}}{\mathrm{Dry}\;\mathrm{weight}}\times 100$$

### 6.11. Histopathology Study

The histopathology of the cornea was evaluated to check the internal damage after treatment with a prepared formulation. The cornea was collected after the permeation study, washed with STF, and stored in formalin solution (10% *v*/*v*.) The cornea was treated with 0.9% NaCl solution taken as a control. Each treated cornea was cleaned with alcohol and mounted with molten paraffin. The cross-section of the cornea was cut and stained with hematoxylin and eosin to evaluate under a high-resolution microscope (BA210m Motic microscope, Selangor Darul Ehsan, Malaysia).

### 6.12. HET-CAM Irritation Study

The in vitro irritation AM-NLopIG, negative control (0.9% NaCl), and positive control (sodium lauryl sulphate, 1% *w/v*) were evaluated by using hen egg chorioallantoic membrane (HET-CAM). The fertilized hen eggs were procured from a poultry farm and incubated for 10 days in a humidity-controlled incubator at 37 ± 0.5 °C/5% RH. On the 10th day, eggs were collected and shells were removed from the air chamber side. The inner membrane was wetted by adding a drop of 0.9% NaCl and then carefully removed by using forceps to visualize developed CAM. Then, 2–3 drops of the AM-NLopIG, NaCl (0.9% *w/v*), and sodium lauryl sulphate (1% *w/v*) were added over CAM and observed for 5 min to check for damage. The scores were given as per the standard scoring scale at different time points, i.e., 0–0.9 for non-irritants, 1–4.9 for weak irritants, 5–8.9 for moderate irritants, and 9–21 for severe irritants [44].

### 6.13. Antimicrobial Study

The activity of AM-NLopIG and eye drop was evaluated by the cup plate method against Staphylococcus aureus (*S. aureus*) and Escherichia coli (*E. coli*). The required quantity of nutrient agar media was weighed, transferred into a conical flask, and dissolved in distilled water. The media was sterilized by autoclave at 121 °C for 15 min. The micro-organisms *S. aureus* and *E. coli* (0.1 mL) were mixed with nutrient agar media and transferred to a sterilized Petri plate under aseptic conditions. The media was kept for solidification in an aseptic area. Using a sterilized borer, 5 mm wells were created and the samples (AM-NLopIG and eye drop) were added to evaluate their efficacy. The Petri plates were kept for 1 h and further incubated at 37 °C for 24 h and 48 h to evaluate the zone of inhibition (ZOI).

## Figures and Tables

**Table 1:** Composition of Azithromycin nano lipid carrier with experimental results.

Code	Independent Variables			Dependent Variables	
	Homogenization Speed (rpm)	Surfactant (%, *w/v*)	Lipid (%, *w/v*)	Particle Size (nm)	Entrapment Efficiency (%)
	A	B	C	Y_1_	Y_2_
AM-NL1	12,000	1.00	3.00	352.2 ± 15.8	56.1 ± 2.5
AM-NL2	20,000	1.00	3.00	228.5 ± 09.2	54.3 ± 2.1
AM-NL3	12,000	3.00	3.00	282.8 ± 13.5	67.9 ± 3.3
AM-NL4	20,000	3.00	3.00	210.3 ± 08.7	63.3 ± 1.9
AM-NL5	12,000	2.00	1.50	260.9 ± 14.2	57.9 ± 1.4
AM-NL6	20,000	2.00	1.50	165.6 ± 6.8	55.7 ± 1.8
AM-NL7	12,000	2.00	4.50	330.9 ± 15.9	80.6 ± 3.6
AM-NL8	20,000	2.00	4.50	220.0 ± 11.2	76.8 ± 2.9
AM-NL9	16,000	1.00	1.50	205.8 ± 9.6	48.8 ± 1.1
AM-NL10	16,000	3.00	1.50	154.7 ± 07.3	56.9 ± 2.1
AM-NL11	16,000	1.00	4.50	257.9 ± 13.8	62.3 ± 2.4
AM-NL12	16,000	3.00	4.50	222.2 ± 11.7	80.9 ± 2.9
* AM-NL13	16,000	2.00	3.00	176.1 ± 12.4	75.4 ± 2.6
* AM-NL14	16,000	2.00	3.00	176.1 ± 12.4	73.4 ± 2.4
* AM-NL15	16,000	2.00	3.00	180.5 ± 11.9	75.4 ± 2.6
* AM-NL16	16,000	2.00	3.00	176.1 ± 12.4	73.4 ± 2.4
* AM-NL17	16,000	2.00	3.00	180.5 ± 11.9	75.4 ± 2.6

* Center point.

**Table 2:** Statistical model fit summary report.

Parameters	Regression Parameters		Models			Model
Particle size			Linear	2FI	Quadratic	Quadratic
	SD		40.76	45.64	2.71	
	R²		0.5929	0.6074	0.9990	
	Adjusted R²		0.4990	−0.3718	0.9978	
	Predicted R²		0.3270	−0.2725	0.9941	
	%CV		-	-	1.22	
	Ade Precision		35,703.02	67,503.77	313.33	
	Lack of fit	F-value	272.57	394.27	0.61	
			Significant	Significant	Non-Significant	
		*p*-value	<0.0001	<0.0001	0.6419	
Entrapment efficiency	SD		6.90	7.66	1.43	Quadratic
	R²		0.6458	0.6640	0.9918	
	Adjusted R²		0.5640	0.4623	0.9812	
	Predicted R²		0.4364	0.0131	0.9013	
	%CV		-	-	2.14	
	Adeq. Precision		−983.49	1722.20	172.20	
	Lack of fit	F-value	68.79	97.85	3.49	
			Significant	Significant	Non-Significant	
		*p*-value	0.0005	0.0003	0.1295	

**Table 3:** Point prediction optimization by Box Behnken design.

Code	Homogenization Speed: Surfactant: Lipid	Actual Value		Predicted Value	
		Y_1_ (nm)	Y_2_ (%)	Y_1_ (nm)	Y_2_ (%)
AM-NL13	16,000:2:3	176.1 ± 12.4	75.4 ± 2.6	178.20	74.7
AM-NLop	16,500:2.25:3	154.1 ± 6.35	78.15 ± 1.9	161.3	79.8

**Table 4:** Physicochemical characterization of in situ gel.

Physicochemical Parameters	AM-G		AM-NLopG	
	Sol State	Gel State	Sol State	Gel State
Viscosity (cps)	131.25 ± 6.23	402.12 ± 5.52	235.34 ± 7.2	495.18 ± 8.7
Cohesiveness (g)	0.8 ± 0.02	0.94 ± 0.01	0.78 ± 0.09	0.86 ± 0.05
Adhesiveness (N-mm)	13.34 ± 0.92	26.23 ± 1.52	14.24 ± 1.1	29.43 ± 1.1
Hardness (N)	3.54 ± 0.32	8.34 ± 0.38	3.21 ± 0.3	8.76 ± 0.5

## Data Availability

Not Applicable.
